# High Plasma Levels of Soluble Intercellular Adhesion Molecule (ICAM)-1 Are Associated with Cerebral Malaria

**DOI:** 10.1371/journal.pone.0084181

**Published:** 2013-12-27

**Authors:** Selorme Adukpo, Kwadwo A. Kusi, Michael F. Ofori, John K. A. Tetteh, Daniel Amoako-Sakyi, Bamenla Q. Goka, George O. Adjei, Dominic A. Edoh, Bartholomew D. Akanmori, Ben A. Gyan, Daniel Dodoo

**Affiliations:** 1 Immunology Department, Noguchi Memorial Institute for Medical Research, College of Health Sciences, University of Ghana, Legon, Accra, Ghana; 2 Department of Animal Biology and Conservation Science, University of Ghana, Legon, Accra, Ghana; 3 Department of Child Health, University of Ghana Medical School, College of Health Sciences, University of Ghana, Legon, Accra, Ghana; Université Pierre et Marie Curie, France

## Abstract

**Background:**

Cerebral malaria (CM) is responsible for most of the malaria-related deaths in children in sub-Saharan Africa. Although, not well understood, the pathogenesis of CM involves parasite and host factors which contribute to parasite sequestration through cytoadherence to the vascular endothelium. Cytoadherence to brain microvasculature is believed to involve host endothelial receptor, CD54 or intercellular adhesion molecule (ICAM)-1, while other receptors such as CD36 are generally involved in cytoadherence of parasites in other organs. We therefore investigated the contributions of host ICAM-1 expression and levels of antibodies against ICAM-1 binding variant surface antigen (VSA) on parasites to the development of CM.

**Methodology/Principal Findings:**

Paediatric malaria patients, 0.5 to 13 years were recruited and grouped into CM and uncomplicated malaria (UM) patients, based on well defined criteria. Standardized ELISA protocol was used to measure soluble ICAM-1 (sICAM-1) levels from acute plasma samples. Levels of IgG to CD36- or ICAM-1-binding VSA were measured by flow cytometry during acute and convalescent states. Wilcoxon sign rank-test analysis to compare groups revealed association between sICAM-1 levels and CM (p<0.0037). Median levels of antibodies to CD36-binding VSA were comparable in the two groups at the time of admission and 7 days after treatment was initiated (p>0.05). Median levels of antibodies to CD36-binding VSAs were also comparable between acute and convalescent samples within any patient group. Median levels of antibodies to ICAM-1-binding VSAs were however significantly lower at admission time than during recovery in both groups.

**Conclusions/Significance:**

High levels of sICAM-1 were associated with CM, and the sICAM-1 levels may reflect expression levels of the membrane bound form. Anti-VSA antibody levels to ICAM-binding parasites was more strongly associated with both UM and CM than antibodies to CD36 binding parasites. Thus, increasing host sICAM-1 levels were associated with CM whilst antibodies to parasite expressing non-ICAM-1-binding VSAs were not.

## Introduction

Malaria infection in man is caused by five species of *Plasmodium*; *Plasmodium falciparum, P. knowlesi, P malariae, P. ovale* and *P. vivax* [1]–[5] and transmitted by the bites of infected female mosquitoes of more than 30 anopheline species. Globally, an estimated 3.3 billion people were at risk of malaria in 2010 with populations living in sub-Saharan Africa having the highest risk of acquiring malaria. Majority of the cases and deaths occur annually in sub-Saharan Africa with children less than five years of age and pregnant women most severely affected [1].

Depending upon the degree of resistance to malaria by an individual, infection could be asymptomatic or manifest clinically as mild or severe malaria. Approximately 2% of all clinical malaria cases in African children results in severe disease that is attributable to *P. falciparum* [6]. This severe clinical manifestation of malaria is responsible for most of the malaria associated deaths [1]. Field isolates of *P. falciparum* that cause severe disease in children tend to express a subset of variant surface antigens (VSAs) with limited recognition by the developing immune system of infants and young children [7], [8]. *Plasmodium falciparum* erythrocyte membrane protein (*Pf*EMP)-1, a component of VSAs, elicits natural immune responses to disease [9], [10]. *Pf*EMP-1 mediates adhesion of infected red blood cells (iRBC) to endothelial receptors such as CD54 or ICAM-1, and CD36. Unlike CD36, ICAM-1 expression on vascular endothelium in the brain is up-regulated during malaria infection [11]–[13]. The degree of ICAM-1 up-regulation is influenced by the parasite and inflammatory cytokines [5], [14]–[17]. Following proteolytic cleavage, ICAM-1 becomes detectable in the blood [18]–[21] and the plasma levels of soluble ICAM-1 (sICAM-1) may perhaps reflect its membrane expression levels. ICAM-1 mediates adhesion of a smaller number of field parasite isolates to vascular endothelium compared to CD36 [11], [22]. The binding of CD36 and ICAM-1 to their respective *Pf*EMP-1 have been shown to be specific and results in a selection of parasites expressing those *Pf*EMP-1 variants [23]. Cytoadherence and subsequent sequestration of iRBC in the brain micro-vessels has been implicated in the pathophysiology of cerebral malaria. Up-regulation of ICAM-1 during infection and the possibility of its increased interaction with parasites expressing the appropriate corresponding *Pf*EMP-1 phenotype may thus be important in the pathogenesis of cerebral malaria. Additionally, naturally induced antibodies that bind to parasite VSAs and have been associated with protection [8]–[10] may prevent parasite interaction with the host receptors, CD36 and ICAM-1, thereby preventing parasite cytoadherence and sequestration. Thus, the expression levels of ICAM-1 in the endothelium, here reflected by the plasma levels of the soluble form of this receptor, and the physiological levels of antibodies against ICAM-1-binding VSA phenotypes of infecting parasites might modulate clinical disease progression. This hypothesis was investigated by analyzing plasma samples from 69 paediatric malaria patients, of which 37 presented with cerebral malaria (CM) and 32 presented with uncomplicated malaria (UM). The data revealed an association of sICAM-1 levels with CM relative to UM. Levels of antibodies against ICAM-1-binding VSA in CM patients were significantly lower at the time of admission than during recovery (*p* = 0.0031). Thus an increase in sICAM-1 levels may be predictive of increased ICAM-1 expression in the brain microvasculature, and hence the development of cerebral complications in malaria. Also, increase in antibody levels to ICAM-1-binding VSA in CM patients after recovery does affirm that CM patients were infected with parasites that express ICAM-1-binding VSAs.

## Materials and Methods

### Malaria patients and sampling

Patients aged 0.5 to13 years who reported to the Department of Child Health, Korle-Bu Teaching Hospital, University of Ghana Medical School, during the 2003–2004 malaria transmission seasons with a diagnosis of *P. falciparum* malaria were enroled in the study after signed informed consent was obtained from their guardians/parents. All participants were febrile (>37.5 °C) at enrolment and had asexual blood parasitaemia of >2500/ul. Strict criteria were used to categorize patients into cerebral malaria and uncomplicated malaria [24]. Briefly, cerebral malaria patients had coma with a score of <3 on the Blantyre scale [25] and with no other attributable cause of cerebral dysfunction while those with uncomplicated malaria were fully conscious and had haemoglobin level of ≥ 8 g/dl.

Blood samples of 5ml were collected into K_3_EDTA vacutainers from study participants during the period of acute infection and for a subset of participants, samples were also taken 7 days later after recovery from disease. Plasma was prepared from blood samples by centrifugation and aliquots stored at –20°C until used for the estimation of the levels of the three biomarkers (sICAM-1 and antibodies to CD36- and ICAM-1-selected parasites).

The study received ethical approval from the Institutional Review Board of the Noguchi Memorial Institute for Medical Research and the Ethics Committee of the University of Ghana Medical School. Malaria treatment was carried out in accordance with the existing institutional guidelines at the time. Patients with UM were treated with a daily dose of chloroquine at 25 mg/kg body weight over 3 days. When treatment failure occurred, 10 mg/kg body weight per day of amodiaquine was administered for 3 days. Patients with cerebral malaria were treated with either amodiaquine syrup via a nasogastric tube at the same dosage as stated or intramuscular quinine sulphate (10 mg/kg body weight every 8 hours). The treatment with intramuscular quinine was changed to syrup at the same dosage when patients regained full consciousness or after 72 h (whichever was earlier) to complete a 7-day course.

### *P. falciparum* lines, immunostaining and flow cytometry

Two genetically distinct laboratory established isolates of *P. falciparum,* generated as described earlier [26], [27] and kindly donated by Professor Lars Hviid of Centre for Medical Parasitology, Copenhagen were used. The FRC_54_ clone binds to host ICAM-1 while the FRC_36_ clone binds to CD36 of the host were used. Parasites were maintained in culture with human type O^+^ RBCs at 4% haematocrit in RPMI 1640 supplemented with Albumax II (GIBCO, USA) and L-glutamine according to previously described standard methods [28]. Cultures were maintained for not more than 14 days in order to minimize parasite mutations and switching to another phenotype. Specific antibodies to ICAM-1 and CD36-selected parasites in the plasma of patients were measured by flow cytometry as described earlier [29], [30]. In brief, RBC infected with late trophozoite and schizont stages of CD36 or ICAM-1-binding parasites were purified (to > 95%) using the magnetic activated cell sorting (MACS) technique. Aliquots of 2×10^5^ purified late stage parasite infected erythrocytes (LSPEs) were labeled with ethidium bromide in a tube to allow for exclusion of uninfected RBCs from the flow cytometric analysis. Labeled LSPEs were sequentially incubated with 5 µl of test plasma followed by 100 µl of 10 µg/ml goat anti-human IgG conjugated to fluorescein isothiocyanate (Vector Laboratories Inc, USA). Samples were washed twice between incubation periods using 2% foetal bovine serum (FBS) in phosphate buffered saline (PBS). A minimum of 5000 LSPEs were acquired on a FACScan flow cytometer (BD Biosciences, San Jose, CA) and the mean fluorescence intensity (MFI) levels of anti-VSA antibodies was analyzed using CellQuest v3.3 (BD Biosciences, San Jose, CA). Pooled hyper-immune Ghanaian and malaria-naïve American plasma samples were used as positive and negative controls respectively. For each parasite line, the test and control plasma samples were assayed on the same day using the same parasite preparation.

### Measurement of plasma sICAM-1 levels

Plasma levels of sICAM-1 were measured by an in-house optimized sandwich ELISA protocol. Nunc microtitre plates weire coated at 50 µl/well with 2.5 µg/ml purified monoclonal sheep anti-human ICAM-1 antibody (MAB 720, R and D Systems, USA) in PBS as diluent, incubated overnight at 4°C and blocked with 300 µl/well of 5% BSA, 0.05% Tween 20 in PBS for 2 h. Recombinant human ICAM-1, serially diluted from 4000 to 31.25 pg/ml in 0.5% BSA, 0.05% Tween 20 in PBS, and plasma samples diluted 500 times in the same buffer, were added at 50 µl/well and incubated for 2 h. This was followed by incubation with 50 µl/well of a 0.1 µg/ml biotinylated anti-human ICAM-1 (BAF 720, R and D Systems, USA) for 2 h, and subsequently by incubation with 50 µl/well of 1 µg/ml streptavidin-peroxidase (Insight Biotechnology Limited, UK) for 25 min. Colour development was achieved by the addition of 100 µl/well TMB substrate (KEM-EM-TEC, Denmark) for 30 min and the colour reaction stopped by addition of 50 µl/well of 0.2M H_2_SO_4_. Optical densities were subsequently read at 450 nm with a 620 nm reference wavelength. Except for the coating step, all incubations were done on a shaker at room temperature and plates were washed with PBS, 0.05% Tween 20 between incubation periods.

### Statistical analysis

The Wilcoxon sign rank-test was used to assess differences between study groups at baseline and for the pair-wise analysis of possible differences in antibody levels between acute and convalescent plasma samples. Antibody levels to CD36- and ICAM-1-binding VSAs were compared in only 15 pairs of acute and convalescent plasma samples since the other patients were lost to follow-up. A logistic regression model was used to find association between the levels of sICAM-1 and anti-VSA antibodies and the development of CM. The R statistical software (version 2.14.0, R development Core Team) was used for all statistical analyses and graphical presentations. *P*<0.05 was considered statistically significant.

## Results

Samples from 37 cerebral malaria patients and 32 uncomplicated malaria patients were analyzed in this study, and a summary of patient characteristics is presented in Table 1. The median ages, haemoglobin levels and parasite densities were not statistically significantly different between the two disease groups.

**Table 1:** Patient characteristics and laboratory data at baseline.

Disease category (n)		Age (years)		Haemoglobin levels (g/dl)	Parasite density (×10^3^ perµl blood)	sICAM-1 levels (µg/ml)#	Anti-VSA (CD36-binding) antibodies (MFI)§	Anti-VSA (CD54-binding) antibodies (MFI)
UM (32)	5 (1–13)		10.20 (8.1–13)		51.61 (3.15–4340.2)	1.5 (0.09–5.21)	60.42 (31.82–104.5)	16.64 (9.74– 35.41)
CM (37)	3.5 (0.5–12)		7.3 (5.2–10.7)		117.81 (4.41–296.17)	2.27 (0.35–6.3)	76.07 (19.3–132.28)	15.8 (11.26–22.8)

Values reported as median (minimum-maximum) and comparisons made using Wilcoxon sign rank test. Antibody levels are expressed as mean fluorescence intensity (MFI). #ICAM-1 levels were statistically significantly higher in CM than in UM (p = 0.0037).

Anti-VSA (CD36-binding) levels were statistically significantly higher in CM than in UM (p = 0.048).

The aim of the study was to find possible associations between the membrane expression of ICAM-1 and the plasma levels of antibodies to CD36- and ICAM-1-binding VSAs on one hand, and the development of cerebral complications in malaria patients. Expression levels of ICAM-1 were assessed by measuring the plasma soluble form of ICAM-1 (sICAM-1) using sandwiched ELISA technique on the assumption that the soluble form reflects its membrane expression levels. Antibody levels to CD36- and ICAM-1-binding VSAs were however measured by a flow cytometric assay. Levels of anti-VSA antibodies were also estimated for the subset (15 samples) of study participants whose plasma samples, taken 7 days after treatment was initiated, were available for comparison. Median levels of soluble ICAM-1 during acute infection were statistically significantly higher in CM patients compared to the median level in UM patients (Table 1, *p* = 0.0037, Wilcoxon sign rank-test). A logistic regression model with the three biomarkers as independent variables confirmed a strong association of sICAM-1 levels with the CM patient group (Table 2). Median levels of antibodies to CD36-selected parasites were also significantly higher in CM patients compared to median levels in UM patients with a borderline p-value (Table 1, *p* = 0.048), but there was no statistically significant difference in median levels of antibodies to ICAM-1-selected parasites between the two study groups (Table 1).

**Table 2:** Logistic regression data for association of sICAM-1 and anti-VSA antibodies with CM.

	crude OR (95%CI)	adj. OR (95%CI)	P (Wald's test)	P (LR-test)
Anti-VSA (CD36)	1.02 (1, 1.04)	1.02 (0.99, 1.04)	0.158	0.147
Anti-VSA (CD54)	0.96 (0.84, 1.08)	0.95 (0.83, 1.09)	0.439	0.428
sICAM-1	1.82 (1.18, 2.82)	1.82 (1.15, 2.87)	0.01	0.005

UM is the reference patient category.

**Figure 1:**
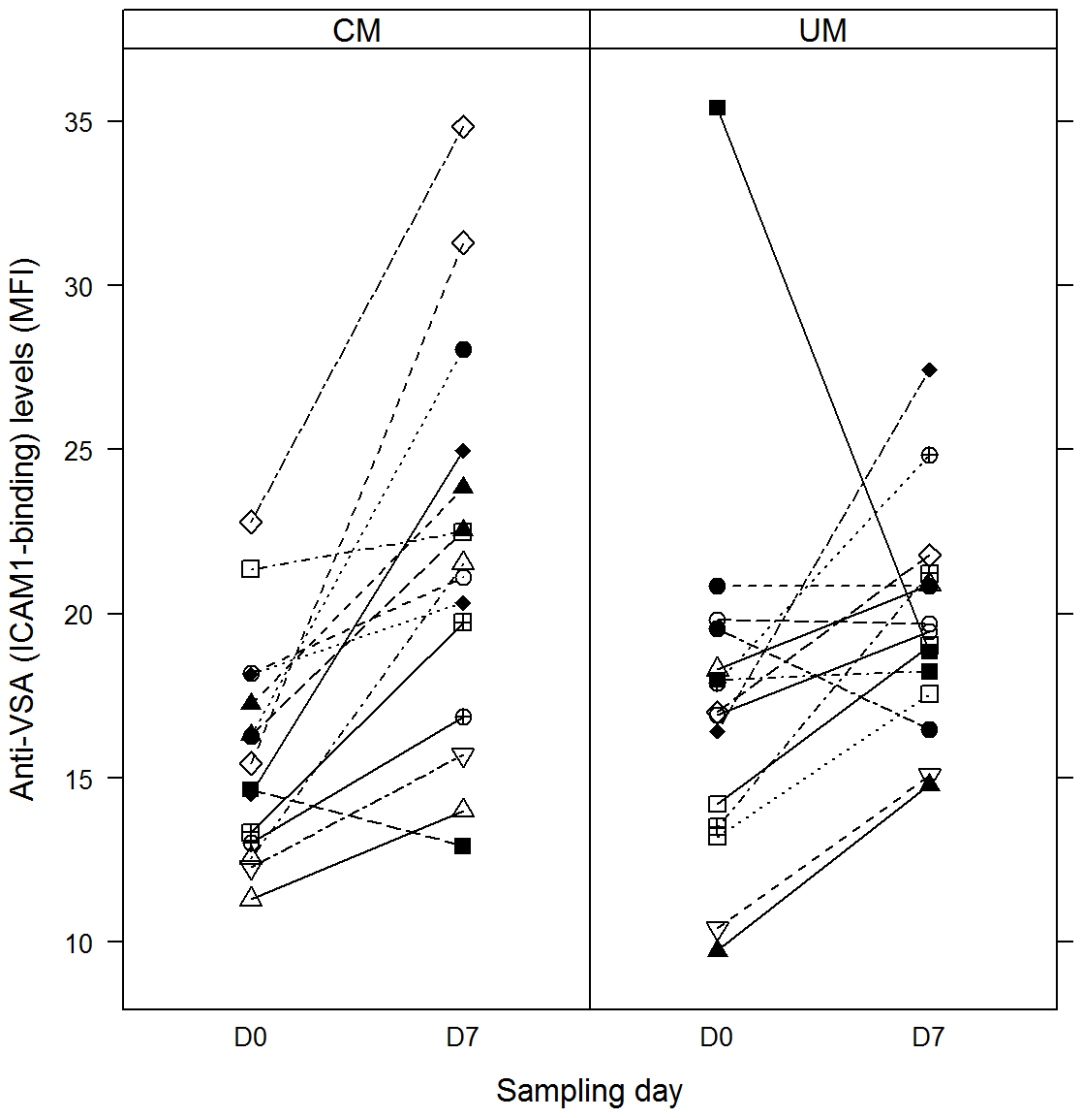
Acute and convalescent levels of antibodies to ICAM-1 selected parasites. Symbols linked by straight line represent anti-VSA antibody levels on days 0 and 7 for individual participants.

**Figure 2:**
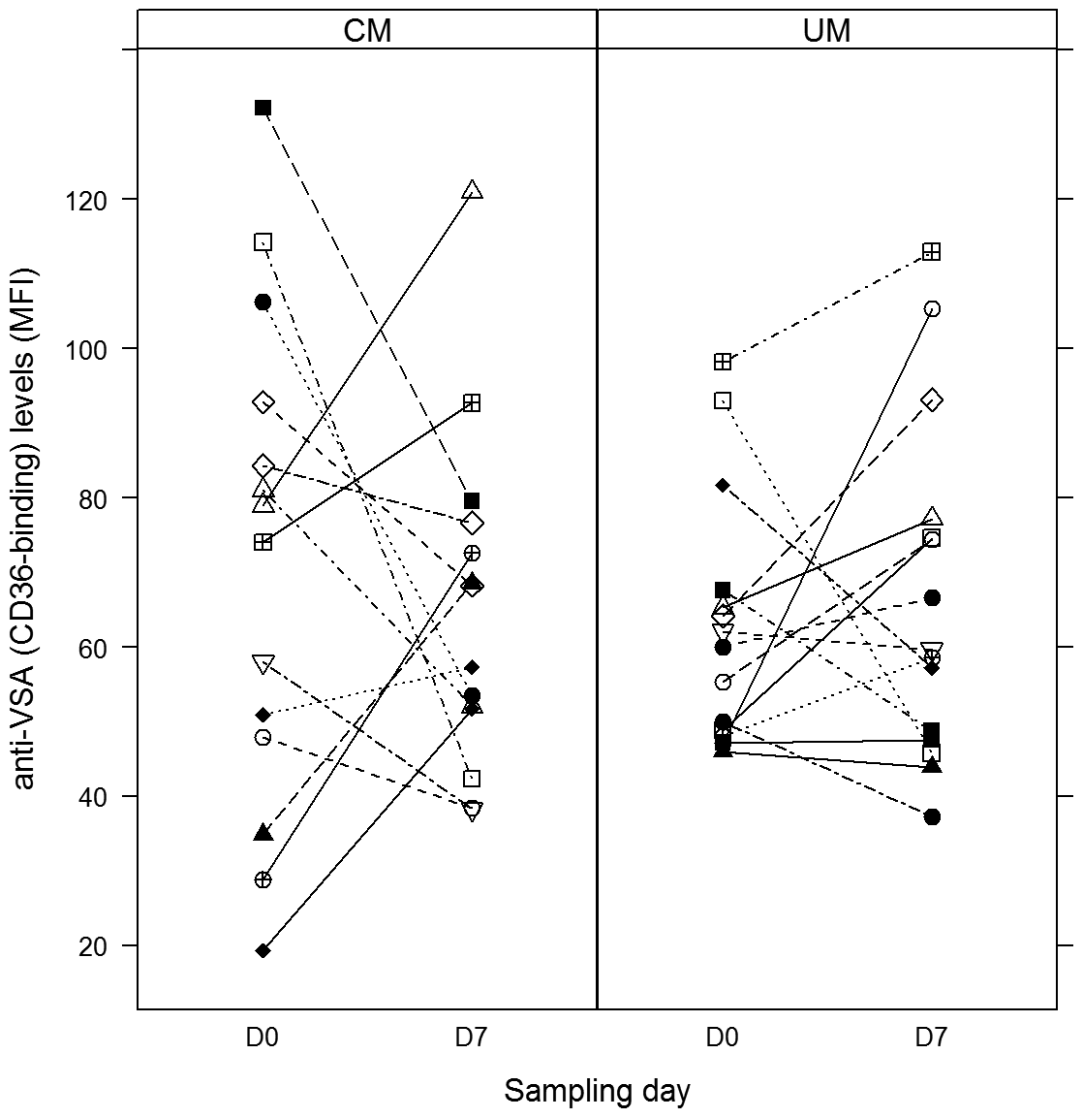
Acute and convalescent levels of antibodies to CD36- selected parasites. Symbols linked by straight line represent anti-VSA antibody levels on days 0 and 7 for individual participants.

Pairwise comparisons of antibody levels to CD36- and ICAM-1-selected parasites were performed using 15 paired acute and convalescent plasma samples from UM and CM patients to assess changes in antibody levels between disease groups and between Day 0 (D0) and Day 7 (D7) plasma samples. While antibody levels to ICAM-1-selected parasites generally increased for most participants on D7 compared to D0 (Figure 1), antibody levels to CD36-selected parasites did not show a general trend of increase in any direction (Figure 2). Thus antibody levels to CD36-selected parasites were not significantly different in study groups, irrespective of whether the pairwise comparison was for the same patient group (D0 versus D7 for UM, D0 versus D7 for CM) or for the same time point sampling (UM versus CM for D0, UM versus CM for D7) (Table 3). Similarly, no significant differences were observed when levels of antibodies to ICAM-1-selected parasites were compared between patient groups for the same time point (UM versus CM for D0, UM versus CM for D7). Pairwise comparison of antibodies to ICAM-1-selected parasites for both UM (p = 0.037) and CM (*p* = 0.0031) groups however showed statistically significant increases on D7 compared to D0 levels (Table 3). Thus, antibody levels to ICAM-1-selected parasites were lower during acute infection but increased significantly after recovery from both uncomplicated and cerebral malaria.

**Table 3:** Comparison of anti-CD36 and anti-CD54 antibody levels between D0 and D7 samples for CM and UM patients.

	Anti-VSA (CD36-binding)			Anti-VSA (CD54-binding)		
Patient group (n)	D0	D7	P value	D0	D7	P value
UM (15)	60.04 (46.02 – 98.22)	59.68 (37.25 – 112.92)	0.81	17 (9.74 – 35.41)	19.47 (14.79 – 27.43)	0.037
CM (15)	76.5 (19.31 – 132.28)	62.77 (38.3 – 121)	0.57	14.44 (11.3 – 22.79)	21.54 (12.92 – 34.83)	0.0031
P value	0.45	0.88		0.41	0.2	

All values reported as median (minimum-maximum). Comparisons were made using the Wilcoxon sign rank- test. Antibody levels are expressed as mean fluorescence intensity (MFI).

## Discussion

During *Plasmodium*’s life cycle in humans, it expresses a number of antigens including *Pf*EMP-1, a component of the VSAs that are expressed on the surface of infected RBCs (iRBCs) by late stage parasites [31]. Adhesion of iRBCs to the host endothelium is mediated by specific host receptor molecules such as intercellular adhesion molecule 1 (ICAM-1), which is the predominant receptor in the brain, chondroitin sulfate A (CSA) in the placenta, and CD36 in other organs. The extent of parasite sequestration has been shown to correlate positively with endothelial ICAM-1 expression [11]–[13], [31]–[37]. The level of soluble ICAM-1 (sICAM-1), detected in circulation following proteolytic cleavage of the membrane form, is also believed to increase during infection [12], [38]. ICAM-1 is also believed to play a role in inflammation as it binds to leukocyte function associated antigen (LFA-1) or integrin Mac-1 expressed on leukocytes and potentiates leukocyte stimulation [39]. *Pf*EMP-1 elicits specific antibody which has been shown to be protective against disease [8]–[10]. In this study, levels of anti-VSA (CD36- and ICAM-1-binding parasites) antibodies in plasma samples from CM patients during the active disease phase (D0) and seven days after treatment was commenced (D7) were quantitatively determined by flow cytometry and compared with the levels in UM control patients. Levels of sICAM-1 in the plasma of both patient groups during the active disease phase were also quantitatively measured by ELISA and compared.

Consistent with earlier reports [40]–[42], this study found that CM patients had higher levels of sICAM-1 compared to UM patients. This may indeed be a result of shedding of the receptor after its increased expression on endothelial cells and leukocytes following endothelial cell activation in CM patients. The increased expression of membrane bound ICAM-1 followed by binding of parasites to it could therefore enhance sequestration of the parasites to cause vascular obstruction and ischaemia as postulated by the mechanical hypothesis of cerebral malaria development [43]–[46]. The ability of sICAM-1 to elicit inflammatory responses could also mediate an increased production of pro-inflammatory cytokines such as TNF-α, whose overproduction has been implicated in pathogenesis of CM as postulated by the inflammatory hypothesis of cerebral malaria development [47]–[50].

For the subgroup of patients whose convalescent (D7) plasma samples were available for analysis, median antibody levels to ICAM-1-binding VSAs were significantly lower in D0 samples compared to D7 samples for both UM and CM patients. Median levels of antibodies to CD36-binding VSA were however not statistically significantly different between D0 and D7 samples for both the UM and CM groups (Table 3). Thus the anti-VSA antibody levels measured in this study were neither predictive of the development of cerebral complications, nor are they predictive of the parasite variants (ICAM1-binding or CD36-binding) that caused disease. These collectively suggest that both UM and CM patients have had previous exposure to both ICAM-1-binding and CD36-binding parasites, and the boosting of antibodies to ICAM-1-binding parasites in both study groups would suggest that the disease causing parasites in both groups might possibly express ICAM-1-binding VSA. The difference in disease outcome between the two groups could then be attributable to the degree of ICAM-1 expression in the presence of parasite expressing ICAM-1 binding VSA and/or other factors aside just infection with ICAM-binding parasites. Such other factors may include amongst others pre-existing anti-malarial immunity, and the time lapse between infection and initiation of appropriate chemotherapy. Under these conditions, a delay in seeking medical care early could allow parasites to multiply enough and sequester in the brain microvasculture and result in a quick progression from UM to CM. Differences in disease outcome could also be attributable to possible multiple parasite variant infections, with the CM group having a greater proportion of ICAM-1-binding parasites as it is not uncommon to find individuals who are infected by different clones of parasite that express different VSAs at the same time [51], [52].

Limitations of this study include our inability to measure sICAM-1 levels in matching convalescent plasma samples for comparison with D0 samples as this would have provided some insight on the expression levels/shedding of the receptor in absence of the infecting parasites and also whether the ICAM-1 levels influence the rate of recovery. In addition, findings in this study would have to be confirmed with much larger sample sizes, although with the current trend of decline in severe malaria cases even in areas that were considered to be highly endemic some years ago, larger sample sizes might be difficult to achieve.

In conclusion, the study found a strong association between elevated levels of plasma sICAM-1 and CM. Anti-VSA antibodies against both CD36- and ICAM-1-selected parasites did not show any association with protection from CM in acute samples. However, the levels of anti-VSA antibodies to ICAM-1-selected parasites, but not CD36-selected parasites, were higher in convalescent samples compared to acute samples for both patient groups, suggesting that there might have been a disproportionate multiplication of parasites expressing ICAM-1-binding VSA compared to those expressing CD-36 binding VSA in both UM and CM patients. This presupposes that infection with ICAM-1 binding parasite variants or pre-existing/induced antibodies to this parasite type may not be predictive of the development of CM, and raises the possibility of the contribution of other factors to CM development. Thus, protection from CM may therefore require contribution of other factors including antibodies to other parasite types. These findings are relevant to our current understanding of the development of complications such as cerebral malaria during *P. falciparum* infection in children
